# In Situ Construction of Ag/TiO_2_/g-C_3_N_4_ Heterojunction Nanocomposite Based on Hierarchical Co-Assembly with Sustainable Hydrogen Evolution

**DOI:** 10.3390/nano10010001

**Published:** 2019-12-18

**Authors:** Rui Geng, Juanjuan Yin, Jingxin Zhou, Tifeng Jiao, Yao Feng, Lexin Zhang, Yan Chen, Zhenhua Bai, Qiuming Peng

**Affiliations:** 1 State Key Laboratory of Metastable Materials Science and Technology, Yanshan University, Qinhuangdao 066004, China; gengrui@ipe.ac.cn (R.G.); pengqiuming@ysu.edu.cn (Q.P.); 2 Hebei Key Laboratory of Applied Chemistry, School of Environmental and Chemical Engineering, Yanshan University, Qinhuangdao 066004, China; jjy1729@163.com (J.Y.); 15833965527@163.com (Y.F.); zhanglexin@ysu.edu.cn (L.Z.); chenyan@ysu.edu.cn (Y.C.); 3 National Engineering Research Center for Equipment and Technology of Cold Strip Rolling, Yanshan University, Qinhuangdao 066004, China; bai_zhenhua@aliyun.com

**Keywords:** heterojunction, Ag/TiO_2_/g-C_3_N_4_, Co-assembly, hydrogen evolution

## Abstract

The construction of heterojunctions provides a promising strategy to improve photocatalytic hydrogen evolution. However, how to fabricate a nanoscale TiO_2_/g-C_3_N_4_ heterostructure and hinder the aggregation of bulk g-C_3_N_4_ using simple methods remains a challenge. In this work, we use a simple in situ construction method to design a heterojunction model based on molecular self-assembly, which uses a small molecule matrix for self-integration, including coordination donors (AgNO_3_), inorganic titanium source (Ti(SO_4_)_2_) and g-C_3_N_4_ precursor (melamine). The self-assembled porous g-C_3_N_4_ nanotube can hamper carrier aggregation and it provides numerous catalytic active sites, mainly via the coordination of Ag^+^ ions. Meanwhile, the TiO_2_ NPs are easily mineralized on the nanotube template in dispersive distribution to form a heterostructure via an N–Ti bond of protonation, which contributes to shortening the interfacial carrier transport, resulting in enhanced electron-hole pairs separation. Originating from all of the above synergistic effects, the obtained Ag/TiO_2_/g-C_3_N_4_ heterogenous photocatalysts exhibit an enhanced H_2_ evolution rate with excellent sustainability 20.6-fold-over pure g-C_3_N_4_. Our report provides a feasible and simple strategy to fabricate a nanoscale heterojunction incorporating g-C_3_N_4_, and has great potential in environmental protection and water splitting.

## 1. Introduction

With the development of semiconductor photocatalysis [1,2,3,4], semiconductor photocatalysts ushered in a glorious era of the photocatalytic water decomposition of H_2_ [5,6,7,8]. Graphitic carbon nitride is currently a prominent semiconductor material that has garnered tremendous attention in academia due to its low cost, environmental friendliness, suitable band structure and impressive physical and chemical stability [9,10,11]. However, the major bottlenecks, such as small specific surface areas and, especially, grievous charge recombination, will generate inferior photocatalytic activity. 

The surface plasmon resonance effect (SPR) is expected to enhance the photocatalytic energy conversion efficiency in the construction of composite photocatalytic materials due to its unique light-substance interaction characteristics, which has attracted much attention in recent years. Metal nanoparticles (Ag, Au, Cu, etc.) with this SPR effect possess excellent electrical conductivity, which can be used as electron acceptors to effectively inhibit the recombination course of photoinduced carriers [12,13,14]. Thus, it is especially suitable for the construction of highly efficient composite photocatalytic materials. For example, Feliz et al. prepared an octahedral molybdenum cluster catalyst for the photocatalytic degradation of water, providing a new strategy for environmentally friendly and low-cost photocatalysts [15]. However, beyond that, as we know, the construction of a heterojunction through coupling g-C_3_N_4_ with various semiconductor materials is still deemed as an effective way to suppress charge recombination and boost photocatalytic activity [16,17,18,19,20]. Given the well-matched band edge offsets and the superior surface activity, titanium dioxide (TiO_2_) stands out among diverse photocatalysts to construct g-C_3_N_4_/TiO_2_ heterostructures. The tacit cooperation between them prompts the charge transfer at the phase interface and optimizes carrier separation efficiency ulteriorly. Consequently, numerous recent research achievements of the g-C_3_N_4_/TiO_2_ nanocomposite have also emerged [21,22,23,24].

Generally, ingenious morphology with structure can inhibit the agglomeration of nanoparticles, and are also conducive to the charge migration, while porous, one-dimensional nanostructures can provide a powerful bow for expanding the specific surface area, accelerating the mobility of charge carriers and inhibiting the recombination of the charge carrier [25,26,27,28]. Unfortunately, the most reported g-C_3_N_4_/TiO_2_ heterostructures so far are achieved by conventional thermal polymerizing of the mixtures of g-C_3_N_4_ (or their precursors, such as sulfonylurea, cyanamide, melamine) and TiO_2_ (or titanium sulfate, butyl titanate), which mostly generates dense and massive particles as well as the badly agglomerated lamellar structure [29,30,31]. Obviously, the large accumulation of g-C_3_N_4_ is not conducive to the formation of a low specific surface area and excellent charge transfer ability. Thus, g-C_3_N_4_/TiO_2_ nanocomposite remains a concern for ameliorating catalytic activity.

Herein, we elaborately designed an Ag/TiO_2_/g-C_3_N_4_ heterostructure by a small-molecular co-assembly of AgNO_3_, Ti(SO_4_)_2_, melamine in hot solution (70 °C) and further calcining. Heterostructures can form spontaneously in the designed solution. All these Ag/TiO_2_/g-C_3_N_4_ heterogenous materials excellently equipped the carrier migration rates with superior photocatalytic H_2_ evolution over bulk g-C_3_N_4_ to couple the morphology control in TiO_2_/g-C_3_N_4_ heterojunctions with Ag SPR effects by in situ co-assembly.

## 2. Materials and Methods

### 2.1. Materials

Melamine (C_3_H_6_N_6_) and titanic sulfate (Ti(SO_4_)_2_) were ordered from Sinopharm Chemical Reagent Co., Ltd. (Shanghai, China). Silver nitrate (AgNO_3_) was purchased from Aladdin Chemicals (Tianjin, China). Polyvinylpyrrolidone (PVP, Mw = 40,000) was provided by Stem Chemicals (Beijing, China). 1-propyl alcohol (99.7%) was gained from J&K Chemicals (Beijing, China).

### 2.2. Synthesis of Ag/TiO_2_/Melamine Heterojunction Nanotubes

Typically, melamine (375 mg), AgNO_3_ (85 mg), Ti (SO_4_)_2_ with different contents (0, 24, 72 and 144 mg) were added into the mixture of aqueous solution and 1-propyl alcohol (99.7%) containing PVP (50 mg), which followed by incubation at 70 °C for 2 h with a continuous magnetic stirrer to in situ construct a series of Ag/TiO_2_/melamine-X nanocomposites (X represents the different dosages of Ti(SO_4_)_2_ precursors, labeled as 0, 1, 2, 3). Finally, the samples were freeze-dried and labeled as Ag/MA, Ag/TiO_2_/MA-1, Ag/TiO_2_/MA-2, Ag/TiO_2_/MA-3, respectively.

### 2.3. Preparation of Ag/TiO_2_/g-C_3_N_4_ Heterogenous Photocatalysts

Under Ar atmosphere, Ag/TiO**_2_**/g-C**_3_**N**_4_**-X (X = 0, 1, 2, 3) was prepared by burning Ag/TiO_2_/Melamine-X precursors to 550 °C for 4 h at a heating rate of 2 °C/min in a tube furnace.

### 2.4. Monitoring of Photocatalytic Hydrogen Evolution Activity

The photocatalytic water-splitting experiments were performed in a 10 mL quartz test tube equipped with a rubber stopper. A 350 W xenon lamp with a 400 nm optical filter was regarded as a light source at a distance of 10 cm away from the photocatalytic reactor. In a typical photocatalytic experiment, 10 mg catalyst was dispersed in 5 mL aqueous solution containing 0.5 mL triethanolamine as a sacrificial agent. Additionally, 0.5 mL potassium tetrachloroplatinate (II) (K_2_PtCl_4_) was added into the hybrid solution with 1 wt % of Pt cocatalyst to photocatalyst. To provide an anaerobic environment, the reaction system was adequately removed from the dissolved oxygen by discharging Ar for 30 min and then sealing with paraffin. In order to keep the sample in suspension status, the photocatalyst was stirred continuously by a magnetic stirrer during the whole experiment. The reaction was monitored for 4 h, and 0.2ml of gas was sampled at intervals of an hour through the septum to analyze by gas chromatography (TRACE 1300, Thermo Fisher Scientific, Waltham, MA, USA, TCD, 5Å molecular sieve column).

### 2.5. Characterization

The morphology of the Ag/TiO_2_/g-C_3_N_4_ composite sample was observed by a transmission electron microscope (TEM, HT7700, High-Technologies Corp., Tokyo, Japan) and a high-resolution transmission electron microscope (HRTEM, JEM-2100F, JEOL, Tokyo, Japan). X-ray diffraction (XRD) was obtained on an X-ray diffractometer equipped with a CuKα X-ray radiation source and a Bragg diffraction apparatus (SMART LAB, Rigaku, Tokyo, Japan). The thermogravimetric (TG) method was carried out under an Ar atmosphere using a NETZSCH STA 409 PC Luxxsi simultaneous thermal analyzer (Netzsch Instruments Manufacturing Co., Ltd., Ahlden, Germany). The FTIR spectrum of the photocatalyst was recorded by Fourier infrared spectroscopy (Thermo Nicolet Corporation, Hemel Hempstead, UK), including KBr flakes. UV-visible diffuse reflectance spectra were examined on a UV-visible spectrophotometer (Hitachi U3010, Tokyo, Japan) using barium sulfate (BaSO_4_) as the substrate. The Brunauer–Emmett–Teller (BET) method was carried out to measure the specific surface areas and pore size distribution on an ASAP 2460 system. X-ray photoelectron spectroscopy (XPS) analyses were achieved via the Thermo Scientific ESCALab 250Xi (Thermo Fisher Scientific, Waltham, MA, USA) with 200 W monochromatic Al Kαradiation. The Hitachi F-4500 fluorescence spectrometer (Hitachi Ltd., Tokyo, Japan) was carried out by monitoring the steady-state fluorescence spectra of the nanocomposites. The time-resolved fluorescence spectrum was acquired on an Edinburgh Instrument F900, for which the above excitation wavelengths are 360 nm.

## 3. Results and Discussions

**Figure 1:**
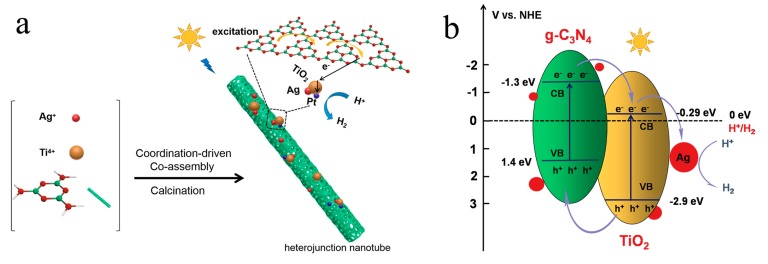
(**a**) Synthetic process and (**b**) the photocatalytic mechanism of Ag/TiO_2_/g-C_3_N_4_ for hydrogen evolution under visible light.

Herein, as shown in Figure 1, we elaborately designed the Ag/TiO_2_/g-C_3_N_4_ heterostructure by a small-molecular co-assembly of AgNO_3_, Ti(SO_4_)_2_, melamine in hot solution (70 °C) and further calcining. Heterostructures can form spontaneously in the designed soup. Specifically, AgNO_3_ can direct the π-stacked structures of melamine into orientationally-organized, one-dimensional nanotubes by a multi-scale synergy of interactions, including the coordination effect and electrostatically-enhanced hydrogen bonds. Meanwhile, TiO_2_ nanoparticles can be easily incubated and self-mineralized on the nanotubes due to the high protonation reactivity of H^+^ from Ti(SO_4_)_2_ with the amine of melamine molecule.

**Figure 2:**
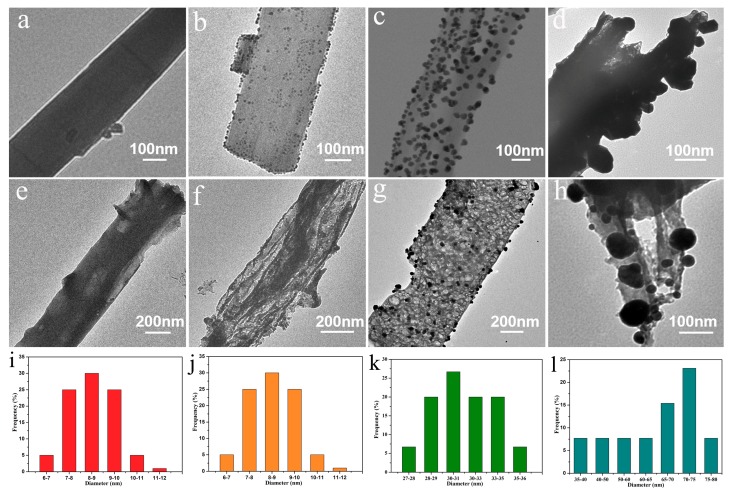
(**a**) Transmission electron microscopy (TEM) images of Ag/MA, (**b**) Ag/TiO_2_/MA-1, (**c**) Ag/TiO_2_/MA-2, (**d**) Ag/TiO2/MA-3, (**e**) Ag/g-C_3_N_4_, (**f**) Ag/TiO_2_/g-C_3_N_4_-1, (**g**) Ag/TiO_2_/g-C_3_N_4_-2, (**h**) Ag/TiO_2_/g-C_3_N_4_-3, (**i**) size histograms of Ag nanoparticles in Ag/g-C_3_N_4_, (**j**) TiO_2_ nanoparticles in Ag/TiO_2_/g-C_3_N_4_-1, (**k**) TiO_2_ nanoparticles in Ag/TiO_2_/g-C_3_N_4_-2, (**l**) TiO_2_ nanoparticles in Ag/TiO_2_/g-C_3_N_4_-3.

The features of as-synthesized samples were researched by TEM. The assembly Ag/MA exhibits nanotube structures with a smooth surface, upon which the mean diameter can reach several hundred nanometers, as shown in Figure 2a. However, only a few nanometers of Ag nanoparticles were found in Ag/MA, as seen in Figure 2i, which may be associated with a limited amount of silver. For all the Ag/TiO_2_/MA nanocomposites, there are numerous TiO_2_ NPs uniformly assembled on the Ag/MA nanotubes as seen in Figure 2b–d. Due to the strong reactivity of H^+^ generated by the hydrolysis of Ti(SO_4_)_2_ under hydrothermal conditions, it can easily be protonated with a large number of basic groups in melamine to form TiO_2_ NPs and in situ anchor simultaneously on the Ag/MA NTS. Interestingly, the mineralized TiO_2_ NPs gradually become larger by increasing the content of Ti(SO_4_)_2_ as showed in Figure 2j–f.

This indicates that the growth of TiO_2_ NPs can be readily manipulated in the assembly process by simply adjusting the precursor mass. After the thermal treatment, all the Ag/TiO_2_/g-C_3_N_4_ present porous structures because of the release of NH3 molecules, as seen in Figure 2e–h.

**Figure 3:**
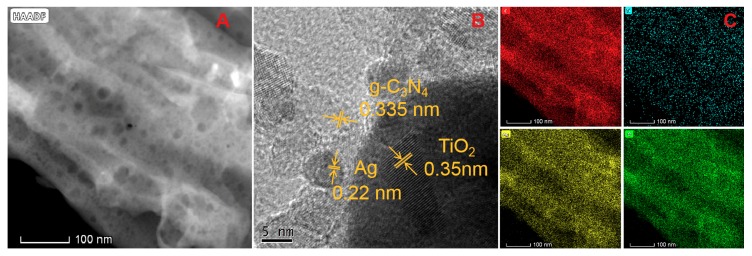
(**A**) Transmission electron microscope (TEM) image and (**B**) high-resolution transmission electron microscope (HRTEM) image of Ag/TiO_2_/g-C_3_N_4_-2. (**C**) Elemental mapping images of the C, Ti, Ag and O elementals.

For the representative morphology of Ag/TiO_2_/g-C_3_N_4_-2 as shown in the Figure 3A TEM image, AgNPs and TiO_2_ NPs of varying sizes are scattered across the g-C_3_N_4_ NTS. The HRTEM image (Figure 3B) shows that the different stacking distances are 0.22, 0.35 and 0.335 nm, corresponding to the (111) lattice spacing of Ag, anatase TiO_2_ (101) crystal plane and (002) lattice plane of g-C_3_N_4_, respectively, which verify the successful co-assembly of Ag, TiO_2_ and g-C_3_N_4_ [32,33]. Additionally, the element mapping images (Figure 3B) of the C, Ti, Ag and O elements in the Ag/TiO_2_/g-C_3_N_4_-2 make it obvious that the Ag element is distributed on the full of the g-C_3_N_4_ NTs surface, but the Ti element is not. Therefore, it further indicates that there is a certain accumulation between Ag and TiO_2_ NPs, which may be beneficial for efficient plasmonic resonant energy transfer between g-C_3_N_4_ NTs and TiO_2_ NPs by the Ag bridge to accelerate charge separation in photocatalytic reactions.

**Figure 4:**
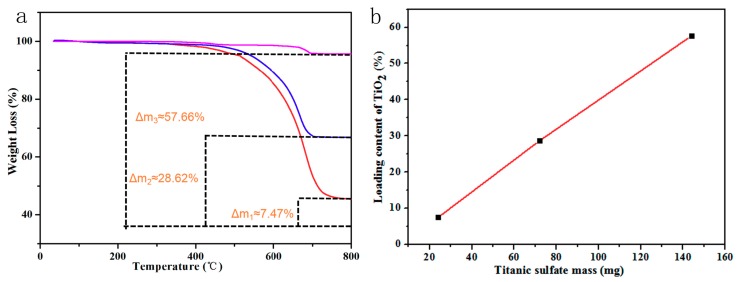
Thermogravimetric analysis (TGA) curves (**a**) of the as-constructed Ag/g-C_3_N_4_, (green); Ag/TiO_2_/g-C_3_N_4_-1, (red); Ag/TiO_2_/g-C_3_N_4_-2, (blue); Ag/TiO_2_/g-C_3_N_4_-3, (pink); (**b**) relationship of loading content of TiO_2_ with Titanic sulfate mass.

Furthermore, TGA was carried out to confirm the mineralization amount of TiO_2_ for the Ag/TiO_2_/g-C_3_N_4_ HNCs from Figure 4. All the samples of Ag/TiO_2_/g-C_3_N_4_ HNCs have significant weight loss at 480–700 °C, which may be correlated with the decomposition of the g-C_3_N_4_ part. So, in other words, the remaining TiO_2_ contents for Ag/TiO_2_/g-C_3_N_4_-x (x = 1, 2, 3) are about 7.47%, 28.62% and 57.66% by subtracting the residue contents of Ag, respectively. Amazingly, the incubated content of TiO_2_ is linearly relying on the mass of Ti(SO_4_)_2_ by feeding in the co-assembly process, as shown in Figure 4b. All the above results adequately prove that it is easy to achieve the controllable surface morphology and depositing amount of TiO_2_ NPs mineralized on g-C_3_N_4_ in the whole assembly evolution.

**Figure 5:**
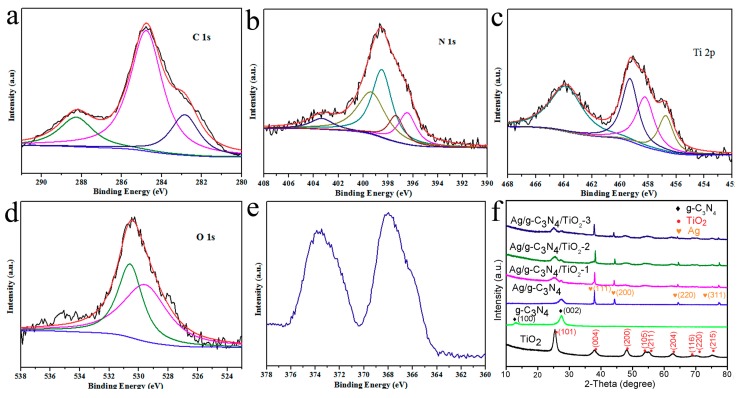
X-ray photoelectron spectroscopy (XPS) spectra of the fabricated Ag/TiO_2_/g-C_3_N_4_-2 (**a**–**e**) showing elements C/N/Ti/O/Ag in the structure; (**f**) X-ray diffraction (XRD) patterns of the g-C_3_N_4_, Ag/g-C_3_N_4_, Ag/TiO_2_/g-C_3_N_4_-1, Ag/TiO_2_/g-C_3_N_4_-2, Ag/TiO_2_/g-C_3_N_4_-3 samples.

X-ray photoelectron spectroscopy (XPS) generally reveals the chemical interfacial bonding state of various composites. The results of Ag/TiO_2_/g-C_3_N_4_-2 HNCs for XPS analysis are displayed in Figure 5a–e. The C 1s spectrum exhibits three different peaks located at 282.8, 284.8 and 288.3 eV are ascribed to the calibration of XPS measurement, sp2 hybridized C atoms in the lattice (C–N bond) with the coordinate N–C=N of the s-triazine rings, respectively. The peaks situated at 397.3, 398.5, 399.4 and 403.4 eV on the N 1s spectrum belong to representative peaks of g-C_3_N_4_, mainly containing sp2-coordinated N from the C=N–C group, sp2 hybridized N in the N–(C)_3_ groups and terminal imino group (C–NH–) in heptazine rings, respectively. Another signal peak located at 396.5 eV, corresponding to a Ti–N bond, suggests that the mineralized TiO_2_ might possess interactions with g-C_3_N_4_. Two peaks at 456.9 and 459.3 eV on the Ti 2p spectrum signify the existence of the N–Ti bond [21,22], also including the Ti 2p_3/2_ peak at 458.2 eV with the Ti 2p_1/2_ peak at 464 eV of two TiO_2_ characteristic bands, respectively. In the O 1s spectrum, the peaks at 529.8 and 530.4 eV originate from the Ti–O bond and O–H bond, respectively. The above analysis suggests that the TiO_2_ NPs are firmly chemically bound to the surface of g-C_3_N_4_ NTs. For the Ag 3d spectrum, the two characteristic peaks of Ag^0^ at the Ag 3d_5/2_ and Ag 3d_3/2_ states can be observed from 367.9 eV and 373.6 eV, respectively. As shown in Table 1, the Ti components obviously increased from 1.25% to 9.73% calculated from the cumulative area of XPS peaks for Ag/TiO_2_/g-C_3_N_4_-1 to Ag/TiO_2_/g-C_3_N_4_-3, while Ag and N components slightly decreased oppositely. In addition, the XRD patterns of TiO_2_ NPs, pure g-C_3_N_4_, Ag/g-C_3_N_4_ NTs, Ag/TiO_2_/g-C_3_N_4_-X HNCs in Figure 5f. All the diffraction peaks for the TiO_2_ NPs can be perfectly attributed to the (101), (004), (200), (105), (211), (204), (116), (220) and (215) lattice planes of anatase phase TiO_2_ (JCPDS, 84-4921) [34]. The distinct peaks located at 13.1° and 27.4° pertain to the tri-s-triazine repeating motif of the (100) plane and the (002) interlayer stacking of pure g-C_3_N_4_, respectively [35]. For Ag/g-C_3_N_4_ NTs, new peaks at around 38.1°, 44.2°, 64.4° and 77.4° are connected with the (111), (200), (220) and (311) planes of the cubic phase of Ag (PDF#04-0783) [36]. Remarkably, the peaks of TiO_2_ become stronger, while the peak intensity of g-C_3_N_4_ gradually becomes weaker with increasing concentrations of the precursor Ti(SO_4_)_2_ for Ag/TiO_2_/g-C_3_N_4_ heterojunction nanocomposites, suggesting that TiO_2_ NPs can be continually assembled onto the Ag/g-C_3_N_4_ NTs [37,38].

**Figure 6:**
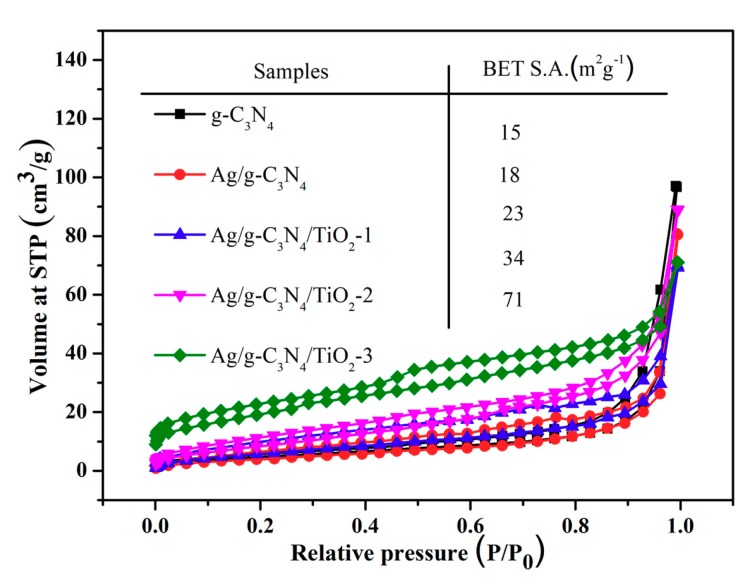
Brunauer–Emmett–Teller (BET) nitrogen adsorption-desorption isotherms of pure g-C_3_N_4_, Ag/g-C_3_N_4_, Ag/TiO_2_/g-C_3_N_4_-1, Ag/TiO_2_/g-C_3_N_4_-2 and Ag/TiO_2_/g-C_3_N_4_-3.

Figure S1 describes the FTIR spectra of all samples. The wide absorption peak around 500 cm^−1^ can be traced to the Ti–O–Ti bond vibration mode of TiO_2_ and the peak intensity enhances with an increasing concentration of Ti(SO_4_)_2_, which accords well with the result of XRD [39]. Several strong peaks ranging from 1200 cm^−1^ to 1600 cm^−1^ belong to the characteristic stretching vibration pattern in the hybrid aromatic CN. The characteristic band centered around 800 cm^−1^ belongs to the breathing modes for the triazine ring [40]. Moreover, the broad peak in the range of 3000 cm^−1^ to 3500 cm^−1^ pertains to the stretching vibration for the NH or NH_2_ groups. Nitrogen adsorption–desorption isotherms were usually used to estimate the specific surface area of the samples [39,40]. As shown in Figure 6, bulk g-C_3_N_4_ possess a relatively small specific surface of 15.344 m^2^g^−1^, while the specific surface areas gradually expand to 18, 23, 34 and 71 m^2^g^−1^ for Ag/g-C_3_N_4_ NTs, Ag/TiO_2_/g-C_3_N_4_-x HNCs (x = 1, 2, 3), respectively [41,42,43,44]. All of the Ag/TiO_2_/g-C_3_N_4_-x HNCs display the existence of mesopores around 12.5 nm, and the gradual narrowing of the peak may be related to the growth of TiO_2_ NPs based on the BJH pore-size distribution curves in Figure S2, which are coincident with the TEM results. These results suggest that the construction of hierarchical self-assembly is beneficial in inhibiting the accumulation of g-C_3_N_4_, and thus enlarging their specific surface area at different scales. Additionally, the porous tubular structures were conducive to the fast transfer of carriers to the surface, and further increase the photocatalytic performance.

**Figure 7:**
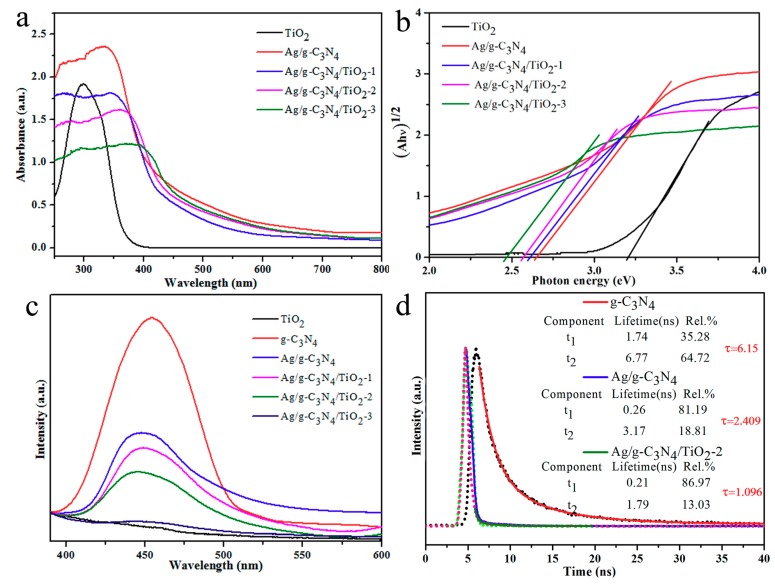
(**a**) UV-vis diffuse reflectance spectra and (**b**) The transformed Kubelka–Munk functions versus the light energy of the as-prepared pureTiO_2_, Ag/g-C_3_N_4_, Ag/TiO_2_/g-C_3_N_4_-1, Ag/TiO_2_/g-C_3_N_4_-2, Ag/TiO_2_/g-C_3_N_4_-3. (**c**) The steady state PL spectra of Ag/TiO_2_/g-C_3_N_4_-x. (**d**) Time resolved PL decay spectra of g-C_3_N_4_, Ag/g-C_3_N_4_, Ag/TiO_2_/g-C_3_N_4_-2.

Generally, the UV-vis diffuse reflectance spectra, transient steady-state spectrometer, enabled us to evaluate the photophysical behaviors of carriers in the photoconductors. The light absorption ability was distinctly revealed by UV-vis absorption spectra. In the visible light region, all the photocatalysts presented the broadened absorption bands and possessed steep curves in the 375–450 nm spectral range (except TiO_2_ NPs), as shown from Figure 7a, which was related to the band gap transition of semiconductors. Due to the optical interaction by combining g-C_3_N_4_ with TiO_2_, the visible light response of the heterogeneous photocatalyst diminishes with the increase of mineralized TiO_2_ NPs. The peak red shift observed from Figure 7a for composite materials resulted from the presence of narrow band gap C_3_N_4_ and probably from the plasmonic properties of silver nanoparticles. In addition, high light absorption capacity does not always mean excellent photocatalytic activity, but also depends on enough low photoinduced electron-hole recombination rates. 

It is known that the peak intensity in steady-state fluorescence spectra was closely related to the detached capability of the photo-generated carrier. According to the steady PL spectra in Figure 7c, all of the Ag/TiO_2_/g-C_3_N_4_-x HNCs present much lower emission peak intensity compared with bulk g-C_3_N_4_, and almost no PL emission was monitored over Ag/TiO_2_/g-C_3_N_4_-3, which is probably because TiO_2_ NPs dominated in the composition. The damped intensity of Ag/TiO_2_/g-C_3_N_4_-x HNCs indicates their better photo-generated electron-hole separation, and thus their recombination was greatly hindered, which could be benefited from the design of heterostructure with Ag SPR effects. Transient fluorescence spectrum of the photocatalysts was measured to ulteriorly inspect the charge carrier’s separation and transfer efficiency from Figure 7d. The emission decay curves of all samples presented exponential attenuation, and could be fitted by the double-exponential formula [30,43]. It is seen that Ag/TiO_2_/g-C_3_N_4_-2 HNCs decayed much faster than pure g-C_3_N_4_ and Ag/g-C_3_N_4_ NTs, which may result in interfacial electron transfer after the construction of a heterojunction. Significantly, the average lifetime of Ag/TiO_2_/g-C_3_N_4_-2 HNCs was about 1.096 ns, which was shorter than the bulk g-C_3_N_4_ (6.15 ns) with Ag/g-C_3_N_4_ NTs (2.409 ns), confirming the relatively better separation of photogenerated carriers. These results further demonstrated the high separation efficiency of interfacial carriers for Ag/TiO_2_/g-C_3_N_4_-2 HNCs, which may promote enhanced hydrogen production activity associated with the formation of good heterostructures [45,46,47].

**Figure 8:**
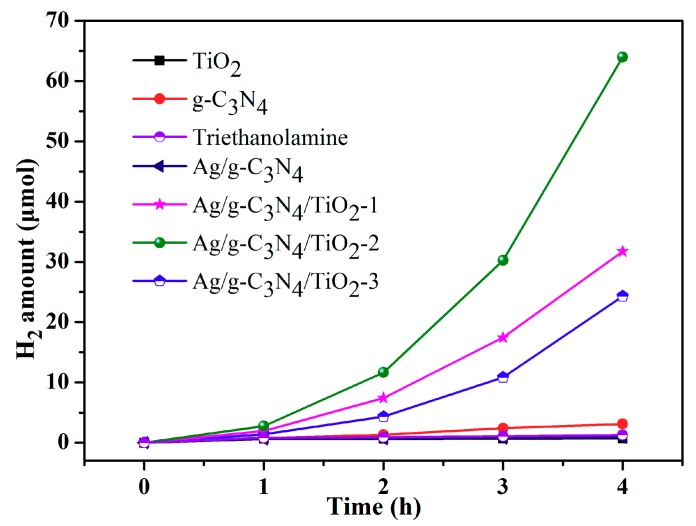
Photocatalytic H_2_ evolution of the samples under visible light irradiation (λ > 400 nm).

The photocatalytic performance of the Ag/TiO_2_/g-C_3_N_4_-x HNCs for the H_2_ production reaction was executed upon solar-simulated light (Figure S3). As given in Figure 8, the bulk g-C_3_N_4_ purely generated about 3.1 μmol of H_2_ after 4 h of illumination. When Ag was introduced to assemble with MA, the calcined sample Ag/g-C_3_N_4_ NTs produced about 24.3 μmol of H_2_ and the value nearly 8-fold more than bulk g-C_3_N_4_, due to Ag SPR effects and the formed porous nanotube structures. 

When incubating AgNO_3_, Ti(SO_4_)_2_ with MA to form a composite by one-pot co-assembly and calcining their precursors, the obtained Ag/TiO_2_/g-C_3_N_4_-1 HNCs generated about 31.75 μmol of H_2_, which is 10.2 times more than pure g-C_3_N_4_, and slightly higher compared with the Ag/g-C_3_N_4_ NTs, benefiting from the constructed Ag/TiO_2_/g-C_3_N_4_ heterojunction. The Ag/TiO_2_/g-C_3_N_4_-2 HNCs presents a substantially enhanced H_2_ evolution amount of 64 μmol, which leads to a 20.6-fold increase on pure g-C_3_N_4_ and much superior to Ag/g-C_3_N_4_ NTs. However, the inconspicuous hydrogen precipitation activity of Ag/TiO_2_/g-C_3_N_4_-3 HNCs may derive from the abundant defects which bring poor photo adsorption and further suppress the carrier transfer process by the introduction of high TiO_2_ content. It should be noted that triethanolamine is a typical sacrificial agent and hole trapping agent often used for photocatalytic hydrogen production. In our present reference system of hydrogen evolution during sacrificial agent photolysis in the absence of a photocatalyst, the obtained generated H_2_ amount shows slight values in comparison with these cases with the photocatalyst, as shown in Figure 8. These consequences mean the significant improvement of photocatalytic activity in samples thanks to the nanoscale structure of g-C_3_N_4_ and the well-established heterojunction in Ag/TiO_2_/g-C_3_N_4_. Specifically, one-dimensional, tube-like g-C_3_N_4_ with the increased specific surface area can effectively avoid carrier aggregation, and provide many more catalytic active sites [48,49,50,51]. Moreover, the heterogeneous structure with nanometer size can reduce the charge diffusion length and effectively facilitate the separation process of charge carriers at the interface, which is also confirmed with the steady-state and transient fluorescence spectrum. In view of the above results, the presently prepared new heterojunction materials provide an exploration of new catalyst fields such as water oxidation and self-assembled composites [52,53,54,55,56,57,58,59,60,61,62,63].

## 4. Conclusions

In summary, we provide a simple and feasible strategy to construct a nanoscale heterojunction model with controllable morphology based on the co-assembly of coordination donor (AgNO_3_), inorganic titanium source (Ti(SO_4_)_2_) and g-C_3_N_4_ precursor (Melamine). The obtained Ag/TiO_2_/g-C_3_N_4_ heterojunction nanocomposite possessing self-organization of multiple kinds of features leads to the superior photocatalytic hydrogen production efficiency and sustainability, which is a 20.6-fold increase compared to pure g-C_3_N_4_. The excellent catalytic performance may have benefited from the following aspects: (1) one-dimensional porous structure, accelerating the separation of charge carriers and providing more reactive sites for photocatalytic process; (2) tight interface, reducing the interfacial charge diffusion migration length and immensely utilizing photogenerated charges; (3) heterostructure, facilitating photogenerated electron-hole pair separation and resulting in improved quantum efficiency with enhanced photocatalytic activity. 

Our work highlights the critical role of the structure nanosizing in the heterojunction system and develops a realistic method to build a platform for potential applications including water oxidation and CO_2_ reduction on the utilization of solar energy.

## Figures and Tables

**Table 1:** Contents (at.%) of prepared composites Ag/TiO_2_/g-C_3_N_4_-X (X = 1, 2, 3) from XPS data.

	C	Ag	N	O	Ti
Ag/TiO_2_/g-C_3_N_4_-1	67.53	3.37	24.43	3.42	1.25
Ag/TiO_2_/g-C_3_N_4_-2	56.69	3.25	22.19	12.95	4.92
Ag/TiO_2_/g-C_3_N_4_-3	40.84	3.06	20.76	25.61	9.73

## Supplementary Materials

The following are available online at https://www.mdpi.com/2079-4991/10/1/1/s1, Figure S1. FT-IR spectra of TiO_2_, Ag/g-C_3_N_4_, Ag/TiO_2_/g-C_3_N_4_-1, Ag/TiO_2_/g-C_3_N_4_-2, Ag/TiO_2_/g-C_3_N_4_-3. Figure S2. The typical BJH pore-size distribution curves (a) Ag/g-C_3_N_4_; (b) Ag/TiO_2_/g-C_3_N_4_-1; (c) Ag/TiO_2_/g-C_3_N_4_-2; (d) Ag/TiO_2_/g-C_3_N_4_-3. Figure S3. TEM image (A) and Elemental mapping images (B–F) of the photodeposited Ag/TiO_2_/g-C_3_N_4_-2, of the C, O, Ti, Pt and Ag elementals.

## References

[1] Huang F., Liu H., Su D. (2017). Graphitized nanocarbon-supported metal catalysts: Synthesis, properties, and applications in heterogeneous catalysis. Sci. China Mater..

[2] Xiao Y.L., Tian C.G., Tian M., Wu A.P., Yan H.J., Chen C.F., Wang L., Jiao Y.Q., Fu H.G. (2018). Cobalt-vanadium bimetal-based nanoplates for efficient overall water splitting. Sci. China Mater..

[3] Liu W.X., Liu Z.Y., Wang G.N., Sun X.M., Li Y.P., Liu J.F. (2017). Carbon coated Au/TiO_2_ mesoporous microspheres: A novel selective photocatalyst. Sci. China Mater..

[4] Li K., Jiao T., Xing R., Zou G., Zhou J., Zhang L., Peng Q. (2018). Fabrication of tunable hierarchical MXene@AuNPs nanocomposites constructed by self-reduction reactions with enhanced catalytic performances. Sci. China Mater..

[5] Bhunia K., Chandra M., Khilari S., Pradhan D. (2019). Bimetallic PtAu alloy nanoparticles-integrated g-C_3_N_4_ hybrid as an efficient photocatalyst for water-to-hydrogen conversion. ACS Appl. Mater. Interfaces.

[6] Wang J., Cong J., Xu H., Wang J., Liu H., Liang M., Gao J., Ni Q., Yao J. (2017). Facile gel-based morphological control of Ag/g-C_3_N_4_ porous nanofibers for photocatalytic hydrogen generation. ACS Sustain. Chem. Eng..

[7] Wang Y., Suzuki H., Xie J., Tomita O., Martin D.J., Higashi M., Kong D., Abe R., Tang J. (2018). Mimicking natural photosynthesis: Solar to renewable H_2_ fuel synthesis by z-scheme water splitting systems. Chem. Rev..

[8] Kampouri S., Stylianou K.C. (2019). Dual-functional photocatalysis for simultaneous hydrogen production and oxidation of organic substances. ACS Catalysis.

[9] Liao G., Gong Y., Zhang L., Gao H., Yang G.-J., Fang B. (2019). Semiconductor polymeric graphitic carbon nitride photocatalysts: The “holy grail” for the photocatalytic hydrogen evolution reaction under visible light. Energy Environ. Sci..

[10] Pei Z., Gu J., Wang Y., Tang Z., Liu Z., Huang Y., Huang Y., Zhao J., Chen Z., Zhi C. (2017). Component matters: Paving the roadmap toward enhanced electrocatalytic performance of graphitic C_3_N_4_-based catalysts via atomic tuning. ACS Nano.

[11] Chong B., Chen L., Han D., Wang L., Feng L., Li Q., Li C., Wang W. (2019). CdS-modified one-dimensional g-C_3_N_4_ porous nanotubes for efficient visible-light photocatalytic conversion. Chin. J. Catal..

[12] Warren S.C., Thimsen E. (2012). Plasmonic solar water splitting. Energy. Environ. Sci..

[13] Clavero C. (2014). Plasmon-induced hot-electron generation at nanoparticle/metal-oxide interfaces for photovoltaic and photocatalytic devices. Nat. Photonics.

[14] Wang S., Gao Y., Miao S., Liu T., Mu L., Li R., Li C. (2017). Positioning the water oxidation reaction sites in plasmonic photocatalysts. J. Am. Chem. Soc..

[15] Feliz M., Atienzar P., Amela-Cortés M., Dumait N., Lemoine P., Molard Y., Cordier S. (2019). Supramolecular anchoring of octahedral molybdenum clusters onto graphene and their synergies in photocatalytic water reduction. Inorg. Chem..

[16] Liu M., Xia P., Zhang L., Cheng B., Yu J. (2018). Enhanced photocatalytic H_2_-production activity of g-C_3_N_4_ nanosheets via optimal photodeposition of Pt as cocatalyst. ACS Sustain. Chem. Eng..

[17] Kumar A., Rana A., Sharma G., Naushad M., Al-Muhtaseb A.H., Guo C., Iglesias-Juez A., Stadler F.J. (2018). High-performance photocatalytic hydrogen production and degradation of levofloxacin by wide spectrum-responsive Ag/Fe_3_O_4_ bridged SrTiO_3_/g-C_3_N_4_ plasmonic nanojunctions: Joint effect of Ag and Fe_3_O_4_. ACS Appl. Mater. Interfaces.

[18] Wang W., Zhao X., Cao Y., Yan Z., Zhu R., Tao Y., Chen X., Zhang D., Li G., Phillips D.L. (2019). Copper phosphide-enhanced lower charge trapping occurrence in graphitic-C_3_N_4_ for efficient noble-metal-free photocatalytic H_2_ evolution. ACS Appl. Mater. Interfaces.

[19] Jiang W., Zong X., An L., Hua S., Miao X., Luan S., Wen Y., Tao F.F., Sun Z. (2018). Consciously constructing heterojunction or direct z-scheme photocatalysts by regulating electron flow direction. ACS Catalysis.

[20] Liu D., Zhang S., Wang J., Peng T., Li R. (2019). Direct z-scheme 2D/2D photocatalyst based on ultrathin g-C_3_N_4_ and WO_3_ nanosheets for efficient visible-light-driven H_2_ generation. ACS Appl. Mater. Interfaces.

[21] You Y., Wang S., Xiao K., Ma T., Zhang Y., Huang H. (2018). Z-scheme g-C_3_N_4_/Bi_4_NbO_8_Cl heterojunction for enhanced photocatalytic hydrogen production. ACS Sustain. Chem. Eng..

[22] Yuan Y.-J., Yang Y., Li Z., Chen D., Wu S., Fang G., Bai W., Ding M., Yang L.-X., Cao D.-P. (2018). Promoting charge separation in g-C_3_N_4_/Graphene/MoS_2_ photocatalysts by two-dimensional nanojunction for enhanced photocatalytic H2 production. ACS Appl. Energy Mater..

[23] Hao R., Wang G., Tang H., Sun L., Xu C., Han D. (2016). Template-free preparation of macro/mesoporous g-C_3_N_4_/TiO_2_ heterojunction photocatalysts with enhanced visible light photocatalytic activity. Appl. Catal. B.

[24] Li J., Zhang M., Li X., Li Q., Yang J. (2017). Effect of the calcination temperature on the visible light photocatalytic activity of direct contact Z-scheme g-C_3_N_4_-TiO_2_ heterojunction. Appl. Catal. B.

[25] Lu Z., Zeng L., Song W., Qin Z., Zeng D., Xie C. (2017). In situ synthesis of C-TiO_2_/g-C_3_N_4_ heterojunction nanocomposite as highly visible light active photocatalyst originated from effective interfacial charge transfer. Appl. Catal. B.

[26] Tan Y., Shu Z., Zhou J., Li T., Wang W., Zhao Z. (2018). One-step synthesis of nanostructured g-C_3_N_4_/TiO_2_ composite for highly enhanced visible-light photocatalytic H_2_ evolution. Appl. Catal. B.

[27] Guo R., Jiao T., Li R., Chen Y., Guo W., Zhang L., Zhou J., Zhang Q., Peng Q. (2018). Sandwiched Fe_3_O_4_/carboxylate graphene oxide nanostructures constructed by layer-by-layer assembly for highly efficient and magnetically recyclable dye removal. ACS Sustain. Chem. Eng..

[28] Wu N., Wang J., Tafen D.N., Wang H., Zheng J.-G., Lewis J.P., Liu X., Leonard S.S., Manivannan A. (2010). Shape-enhanced photocatalytic activity of single-crystalline anatase TiO_2_ (101) nanobelts. J. Am. Chem. Soc..

[29] Wu K., Zhu H., Liu Z., Rodriguez-Cordoba W., Lian T. (2012). Ultrafast charge separation and long-lived charge separated state in photocatalytic CdS-Pt nanorod heterostructures. J. Am. Chem. Soc..

[30] Gu W., Lu F., Wang C., Kuga S., Wu L., Huang Y., Wu M. (2017). Face-to-face interfacial assembly of ultrathin g-C_3_N_4_ and anatase TiO_2_ nanosheets for enhanced solar photocatalytic activity. ACS Appl. Mater. Interfaces.

[31] Wei S., Zhang F., Zhang W., Qiang P., Yu K., Fu X., Wu D., Bi S., Zhang F. (2019). Semiconducting 2D Triazine-cored covalent organic frameworks with unsubstituted olefin linkages. J. Am. Chem. Soc..

[32] Yan J., Wu H., Chen H., Zhang Y., Zhang F., Liu S.F. (2016). Fabrication of TiO_2_/C_3_N_4_ heterostructure for enhanced photocatalytic Z-scheme overall water splitting. Appl. Catal. B.

[33] Li K., Huang Z., Zeng X., Huang B., Gao S., Lu J. (2017). Synergetic effect of Ti^3+^ and Oxygen doping on enhancing photoelectrochemical and photocatalytic properties of TiO_2_/g-C_3_N_4_ heterojunctions. ACS Appl. Mater. Interfaces.

[34] Zhou X., Shao C., Li X., Wang X., Guo X., Liu Y. (2018). Three dimensional hierarchical heterostructures of g-C_3_N_4_ nanosheets/TiO_2_ nanofibers: Controllable growth via gas-solid reaction and enhanced photocatalytic activity under visible light. J. Hazard. Mater..

[35] Liu Q.X., Ai L.H., Jiang J. (2018). MXene-derived TiO_2_@C/g-C_3_N_4_ heterojunctions for highly efficient nitrogen photofixation. J. Mater. Chem. A.

[36] Ou M., Wan S.P., Zhong Q., Zhang S.L., Song Y., Guo L.N., Cai W., Xu Y.L. (2018). Hierarchical Z-scheme photocatalyst of g-C_3_N_4_@Ag/BiVO_4_ (040) with enhanced visible-light-induced photocatalytic oxidation performance. Appl. Catal. B.

[37] Tang L., Feng C.Y., Deng Y.C., Zeng G.M., Wang J.J., Liu Y.N., Peng H.P., Wang J.J. (2018). Enhanced photocatalytic activity of ternary Ag/g-C_3_N_4_/NaTaO_3_ photocatalysts under wide spectrum light radiation: The high potential band protection mechanism. Appl. Catal. B.

[38] Wang Y.B., Zhao X., Cao D., Wang Y., Zhu Y.F. (2017). Peroxymonosulfate enhanced visible light photocatalytic degradation bisphenol A by single-atom dispersed Ag mesoporous g-C_3_N_4_ hybrid. Appl. Catal. B.

[39] Li K., Gao S., Wang Q., Xu H., Wang Z., Huang B., Dai Y., Lu J. (2015). In-situ-reduced synthesis of Ti^3+^ self-doped TiO_2_/g-C_3_N_4_ heterojunctions with high photocatalytic performance under LED light irradiation. ACS Appl. Mater. Interfaces.

[40] Pan J.Q., Dong Z.J., Wang B.B., Jiang Z.Y., Zhao C., Wang J.J., Song C.S., Zheng Y.Y., Li C.R. (2019). The enhancement of photocatalytic hydrogen production via Ti^3+^ self-doping black TiO_2_/g-C_3_N_4_ hollow core-shell nano-heterojunction. Appl. Catal. B.

[41] Mao M.L., Yan F.L., Cui C.Y., Ma J.M., Zhang M., Wang T.H., Wang C.S. (2017). Pipe-wire TiO_2_-Sn@Carbon nanofibers paper anodes for lithium and sodium ion batteries. Nano Lett..

[42] Lin L., Ye P., Cao C., Jin Q., Xu G.-S., Shen Y.-H., Yuan Y.-P. (2015). Rapid microwave-assisted green production of a crystalline polyimide for enhanced visible-light-induced photocatalytic hydrogen production. J. Mater. Chem. A.

[43] Zhang Z., Huang J., Zhang M., Yuan L., Dong B. (2015). Ultrathin hexagonal SnS_2_ nanosheets coupled with g-C_3_N_4_ nanosheets as 2D/2D heterojunction photocatalysts toward high photocatalytic activity. Appl. Catal. B.

[44] Chen K., Chai Z., Li C., Shi L., Liu M., Xie Q., Zhang Y., Xu D., Manivannan A., Liu Z. (2016). Catalyst-free growth of three-dimensional graphene flakes and graphene/g-C_3_N_4_ composite for hydrocarbon oxidation. ACS Nano.

[45] Zeng L., Lu Z., Li M., Yang J., Song W., Zeng D., Xie C. (2016). A modular calcination method to prepare modified N-doped TiO_2_ nanoparticle with high photocatalytic activity. Appl. Catal. B.

[46] Fontelles-Carceller O., Munoz-Batista M.J., Fernandez-Garcia M., Kubacka A. (2016). Interface effects in sunlight-driven Ag/g-C_3_N_4_ composite catalysts: Study of the toluene photodegradation quantum efficiency. ACS Appl. Mater. Interfaces.

[47] Wei X., Shao C., Li X., Lu N., Wang K., Zhang Z., Liu Y. (2016). Facile in situ synthesis of plasmonic nanoparticles-decorated g-C_3_N_4_/TiO_2_ heterojunction nanofibers and comparison study of their photosynergistic effects for efficient photocatalytic H_2_ evolution. Nanoscale.

[48] Yin J., Zhan F., Jiao T., Deng H., Zou G., Bai Z., Zhang Q., Peng Q. (2019). Highly efficient catalytic performances of nitro compounds via hierarchical PdNPs-loaded MXene/polymer nanocomposites synthesized through electrospinning strategy for wastewater treatment. Chin. Chem. Lett..

[49] Zhao J., Yin J., Zhong J., Jiao T., Bai Z., Wang S., Zhang L., Peng Q. (2020). Facile preparation of a self-assembled artemia cyst shell-TiO_2_-MoS_2_ porous composite structure with highly efficient catalytic reduction of nitro compounds for wastewater treatment. Nanotechnology.

[50] Zhu J., Wang R., Geng R., Zhang X., Wang F., Jiao T., Yang J., Bai Z., Peng Q. (2019). A facile preparation method for new two-component supramolecular hydrogels and their performances in adsorption, catalysis, and stimuli-response. RSC Adv..

[51] Wang C., Yin J., Han S., Jiao T., Bai Z., Zhou J., Zhang L., Peng Q. (2019). Preparation of palladium nanoparticles decorated polyethyleneimine/polycaprolactone composite fibers constructed by electrospinning with highly efficient and recyclable catalytic performances. Catalysts.

[52] Jin Q., Chen S., Sang Y., Guo H., Dong S., Han J., Chen W., Yang X., Li F., Duan P. (2019). Circularly polarized luminescence of achiral open-shell pi-radicals. Chem. Commun..

[53] Jin Q., Chen S., Jiang H., Wang Y., Zhang Li., Liu M. (2018). Self-assembly of amphiphilic schiff base and selectively turn on circularly polarized luminescence by Al^3+^. Langmuir.

[54] Zhang Y., Yang D., Han J., Zhou J., Jin Q., Liu M., Duan P. (2018). Circularly polarized luminescence from a pyrene-cyclodextrin supra-dendron. Langmuir.

[55] Jin Q., Li J., Zhang Li., Fang S., Liu M. (2015). Reactive organogels based on isoxazole esters: Alkali metal ions selective gelation and crystallization. CrystEngComm.

[56] Jin Q., Li J., Li X., Zhang Li., Fang S., Liu M. (2014). Function and application of supramolecular gels chiral molecular recognition and asymmetric catalysis. Prog. Chem..

[57] Li H., Yin J., Meng Y., Liu S., Jiao T. (2020). Nickel/Cobalt-containing polypyrrole hydrogel-derived approach for efficient ORR electrocatalyst. Colloid Surf. A.

[58] Ma K., Wang R., Jiao T., Zhou J., Zhang L., Li J., Bai Z., Peng Q. (2020). Preparation and aggregate state regulation of co-assembly graphene oxide-porphyrin composite Langmuir films via surface-modified graphene oxide sheets. Colloid Surf. A.

[59] Meng Y., Yin J., Jiao T., Bai J., Zhang L., Su J., Liu S., Bai Z., Cao M., Peng Q. (2020). Self-assembled copper/cobalt-containing polypyrrole hydrogels for highly efficient ORR electrocatalysts. J. Mol. Liq..

[60] Hou N., Wang R., Geng R., Wang F., Jiao T., Zhang L., Zhou J., Bai Z., Peng Q. (2019). Facile preparation of self-assembled hydrogels constructed by poly-cyclodextrin and poly-adamantane as highly selective adsorbents for wastewater treatment. Soft Matter.

[61] Ma K., Chen W., Jiao T., Jin X., Sang Y., Yang D., Zhou J., Liu M., Duan P. (2019). Boosting circularly polarized luminescence of small organic molecules via multi-dimensional morphology control. Chem. Sci..

[62] Feng Y., Wang R., Yin J., Zhan F., Chen K., Jiao T., Zhou J., Zhang L., Peng Q. (2020). Facile synthesis of Cu_2_O nanoparticle-loaded carbon nanotubes composite catalysts for reduction of 4-nitrophenol. Curr. Nanosci..

[63] He Y., Wang R., Jiao T., Yan X., Wang M., Zhang L., Bai Z., Zhang Q., Peng Q. (2019). Facile preparation of self-assembled layered double hydroxide-based composite dye films as new chemical gas sensors. ACS Sustain. Chem. Eng..

