# Supplementary material for: In Situ Construction of Ag/TiO2/g-C3N4 Heterojunction Nanocomposite Based on Hierarchical Co-Assembly with Sustainable Hydrogen Evolution
Source: Nanomaterials (Basel). 2019 Dec 18;10(1):1. doi: 10.3390/nano10010001 (PMC7022471; doi:10.3390/nano10010001)
Supplement: Supplementary file 1 [file nanomaterials-10-00001-s001.pdf]

Supporting information

In Situ Construction of $Ag/TiO_2/g\text{-}C_3N_4$ Heterojunction Nanocomposite Based on Hierarchical Co-Assembly with Sustainable Hydrogen Evolution

Rui Geng [1,2], Juanjuan Yin [2], Jingxin Zhou [2,*], Tifeng Jiao [1,2,*], Yao Feng [2], Lexin Zhang [2], Yan Chen [2], Zhenhua Bai [3] and Qiuming Peng [1]

1 State Key Laboratory of Metastable Materials Science and Technology, Yanshan University, Qinhuangdao 066004, China; gengrui@ipe.ac.cn (R.G.); pengqiuming@ysu.edu.cn (Q.P.)

2 Hebei Key Laboratory of Applied Chemistry, School of Environmental and Chemical Engineering, Yanshan University, Qinhuangdao 066004, China; jjy1729@163.com (J.Y.); 15833965527@163.com (Y.F.); zhanglexin@ysu.edu.cn (L.Z.); chenyan@ysu.edu.cn (Y.C.)

3 National Engineering Research Center for Equipment and Technology of Cold Strip Rolling, Yanshan University, Qinhuangdao 066004, China; bai_zhenhua@aliyun.com

* Correspondence: zhoujingxin@ysu.edu.cn (J.Z.); tfjiao@ysu.edu.cn (T.J.); Tel.: +86-335-806-1569 (J.Z.); +86-335-805-6854 (T.J.)

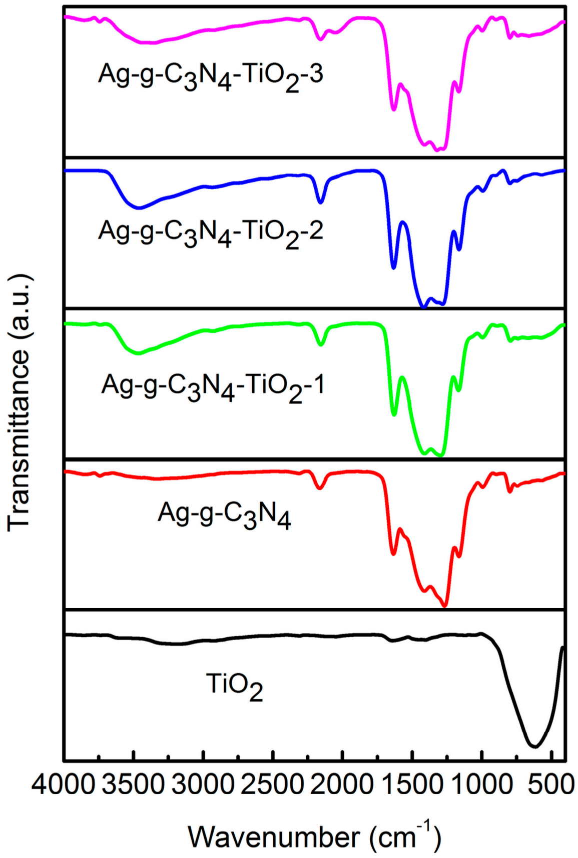

Figure S1. FT-IR spectra of TiO_2, $Ag/g\text{-}C_3N_4$, $Ag/TiO_2/g\text{-}C_3N_4$-1, $Ag/TiO_2/g\text{-}C_3N_4$-2, $Ag/TiO_2/g\text{-}C_3N_4$-3.

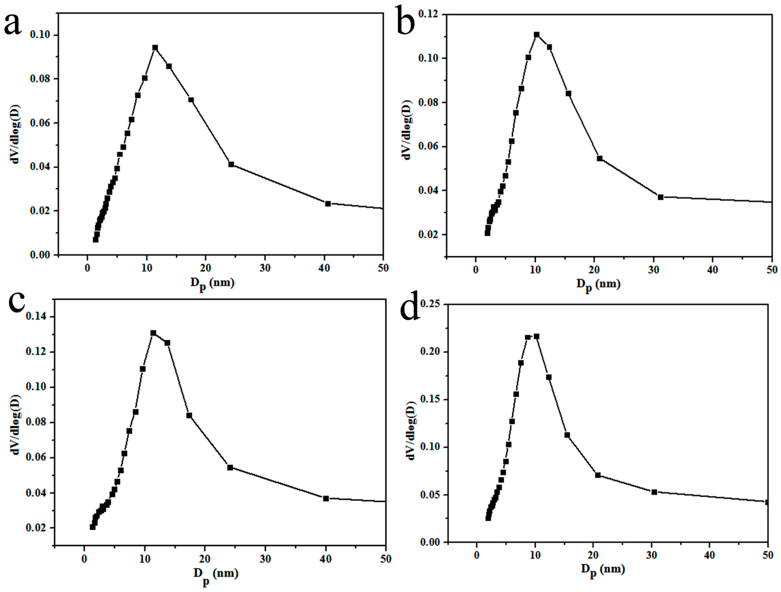

Figure S2. The typical BJH pore-size distribution curves: (a) $Ag/g-C_3N_4$; (b) $Ag/TiO_2/g-C_3N_4$-1; (c) $Ag/TiO_2/g-C_3N_4$-2; (d) $Ag/TiO_2/g-C_3N_4$-3.

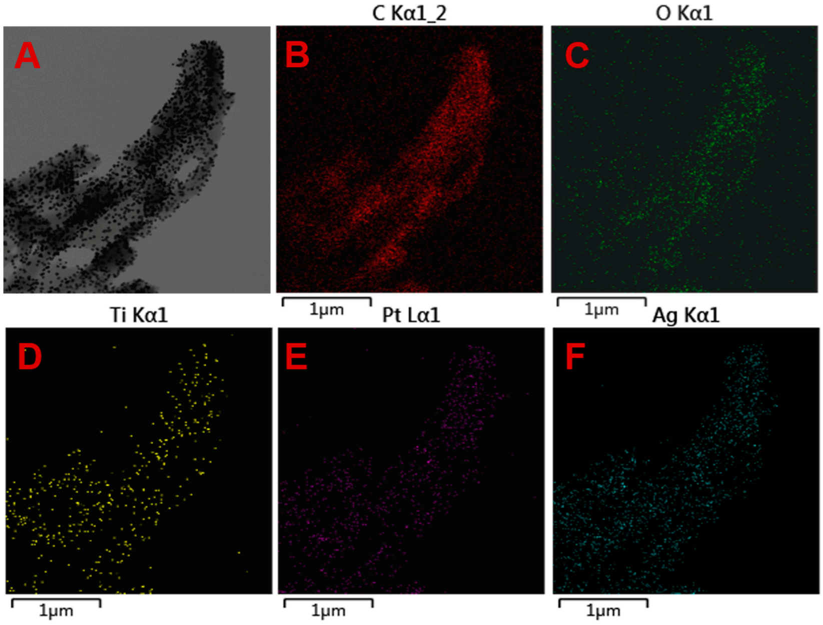

Figure S3. TEM image (A) and Elemental mapping images (B-F) of the photodeposited $Ag/TiO_2/g-C_3N_4$-2.
